# Supplementary material for: Scale-Dependent Dielectric Properties in BaZr0.05Ti0.95O3 Ceramics
Source: Materials (Basel). 2020 Oct 1;13(19):4386. doi: 10.3390/ma13194386 (PMC7579612; doi:10.3390/ma13194386)
Supplement: Supplementary file 1 [file materials-13-04386-s001.pdf]

materials

MDPI

Supplementary Materials

Scale Dependent Dielectric Properties in $BaZr_{0.05}Ti_{0.95}O_3$ Ceramics

1. Processing Parameters

Table S1. Sintering parameters, grain size and relative density for $BaZr_{0.05}Ti_{0.95}O_3$ ceramics.

T_{sint}	GS (µm)	Relative Density (%)
1150 °C/4 h	0.45	72
1175 °C/4 h	1.07	83
1200 °C/4 h	1.22	81
1150 °C/12 h	1.31	73
1200 °C/2 h	1.44	94
1300°C/2 h	1.56	97
1200 °C/2 h + 1300 °C/2 h	5.75	95
1300 °C/3 h	12.15	97
1300 °C/4 h	12.65	97
1350 °C/2 h	50.28	97
1400 °C/2 h	83.76	97
1450 °C/2 h	96.22	97
1500 °C/2 h	114.32	97
1500 °C/4 h	116.08	97
1500 °C/8 h	134.24	97
1500 °C/24 h	137.82	97

2. Temperature Dependence of Dielectric Permittivity

The evolution with temperature of real part of permittivity was presented in manuscript up to 150 °C (Figure 2) and size dependent permittivity was discussed for two temperatures: 25 °C (in orthorhombic phase) and 70 °C (in tetragonal phase). To eliminate the contribution of domain patterns to dielectric permittivity and to identify the role of grain boundary dilution, the dependence of permittivity with grain size must be represented also in paraelectric phase, both as measured and in corrected form. Taking into account that the Curie temperature of investigated ceramics is ~105 °C (with a broad transition for ceramics with small grains) our highest measuring temperature (150 °C) is too close to phase transition and some polar nanoregions may still exist [1]. Therefore, the measured data were extrapolated to 200 °C using Curie-Weiss law (Figure S1) and the grain size (GS) dependences of the dielectric permittivity for three temperatures (25 °C—orthorhombic, 70 °C—tetragonal and 200 °C—cubic) were represented in the inset of Figure S1.

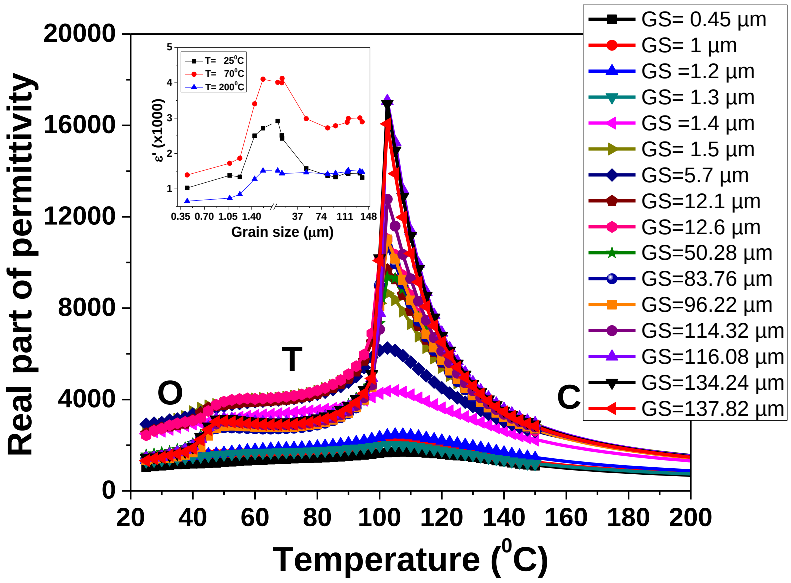

Figure S1. The measured temperature dependence of real part of permittivity of BZT ceramics with different GS extrapolated up to 200 °C. Inset: GS dependence of permittivity at three selected temperatures.

A difference of ~60% between the permittivity of GS = 0.45 μm and the permittivity values of ceramics with GS larger than 1.5 μm, even for the highest temperature, can be observed.

The temperature evolution of the corrected permittivity, according to the di-phase simulations presented in the manuscript, is presented in Figure S2.

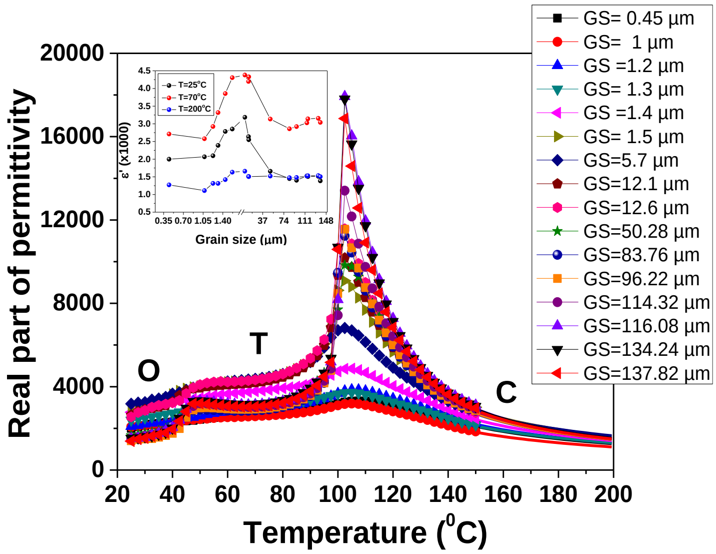

Figure S2. The corrected temperature dependence of permittivity of BZT ceramics with different GS and extrapolated up to 200 °C. Inset: GS dependence of dielectric permittivity at three selected temperatures.

It can be observed that, for the ceramics with larger GS (> 1.4 μm), the permittivity values superimpose for temperatures larger than 180 °C, while for ceramics with fine grains some difference still exist. From the inset of Figure S2 it can also be observed that the effect of grain size is reduced in paraelectric state for ceramics with GS larger than 1.4 μm and only for the fine ceramics (GS = 0.45 μm and GS = 1 μm) a small decrease of relative permittivity (~20%) is present, which can be assigned to the dilutions effect created by grain boundaries.

3. Investigation of the Role of Grain Boundaries in Finite Element Simulations

A real ferroelectric ceramic is not a perfectly continuous medium and, in an ideal simulation approach, it should be modeled as a composite with grain bulk and grain boundaries [2]. However, in the case of porous ferroelectric ceramics with sub-micrometric GSs, it is very difficult to describe tri-phase composites due to several meshing issues and important computational limitations. For example, in the 3D FEM approach presented in the manuscript, we have used a very large number of elements (24 million of tetrahedra), but even in this condition we were not able to simulate the role of the grain boundaries because the average size of the elements (minimum 60 nm) is still very high when compared with grain boundaries thickness. However, the role of the grain boundaries can be qualitatively discussed in the case of 2D FEM simulations, where the elements are much more efficiently distributed in a 2D surface.

The following study was performed to elucidate how important the boundaries are and to what extent a di-phase composite model can accurately describe a real porous ceramic. A 2D FEM approach similar to the one proposed in Ref. [3] was implemented to describe the effective permittivity of 3 types of systems: (a) dense ceramic systems (di-phase composites with grain bulk and grain boundaries), (b) porous ceramic systems (tri-phase composites with grain bulk, grain boundaries and a circular pore located in the center of the system) and (c) simplified porous systems (di-phase composite with ceramic bulk and a circular pore located in the center of the system). The 2D ceramic systems were generated by Potts models and the procedure is described in detail elsewhere [3,4]. The following numerical constants were considered in simulations: size of the system—470 a.u., thickness of the boundaries—3 a.u., diameter of the pore—188 a.u, permittivity of the grain bulk—1000, permittivity of the grain boundaries—100, permittivity of the air pore—1. The only variables considered in these simulations are the GS (used in the case of the first and the second type of systems) and the effective permittivity of the ceramic (used in the case of the third type of systems and computed from the first type of systems). Therefore, the numerical values used in these simulations are not intended to describe a particular ferroelectric system, but they are used only to derive a general comparison between the di-phase and tri-phase approaches in FEM, when simulating the dielectric properties of porous ferroelectric ceramics.

The first simulations (Figure S3) correspond to a GS of 140 a.u.

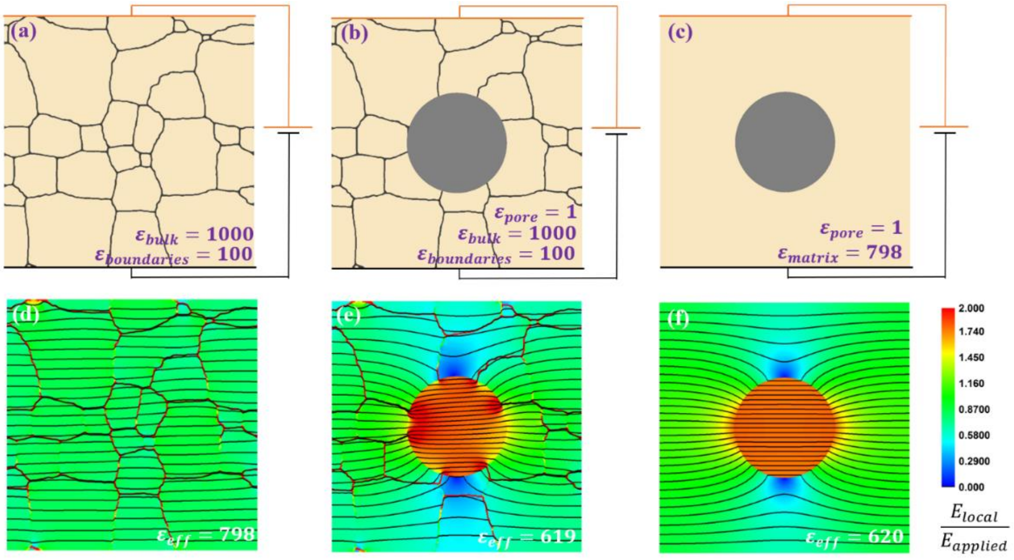

Figure S3. 2D FEM simulations for 3 ceramic systems with GS = 140 a.u.: (**a**,**d**) a dense ceramic formed by grain bulk and grain boundaries, (**b**,**f**) a porous system (tri-phase model) in which grain bulk and grain boundaries were explicitly defined and (**c**,**e**) a porous system (di-phase model) in which grains and boundaries were defined as a single phase characterized by the equivalent permittivity computed by FEM for the dense system (**a**). The local field images (**d**–**f**) are represented in color scale and the black lines are isopotential.

The calculated effective permittivity of the dense ceramic (Figure S3a,d) is of 798, which is 20% smaller than the permittivity of the bulk. The average local field value on bulk is close to the applied one (green color in Figure S3d) which shows that, in this case, the impact of grain boundaries is still reduced. The local field images calculated for the porous ferroelectric systems (Figure S3e,f) by the two approaches (tri-phase and di-phase composite model) present similar characteristics, even if they are not identical: relatively homogeneous local field inside the pore, lower values of the local field on the bulk close to the horizontal ceramic-pore interfaces and higher values of the local field on bulk close to the vertical ceramic-pore interfaces. More remarkable is the fact that the effective permittivity values of the entire porous system calculated by the two approaches are similar: 619 in the case of the tri-phase model and 620 in the case of the di-phase model.

For intermediate values of grain size (Figure S4), an increasing influence of the grain boundaries is noticed: the effective permittivity of the dense ceramic decreases with more than 30% from the permittivity of the bulk, the local field inside some grains tends to be reduced (blue color in Figure S4d) and the local field images of the porous structure do not present the same similarities like in the previous case, especially on the bulk component.

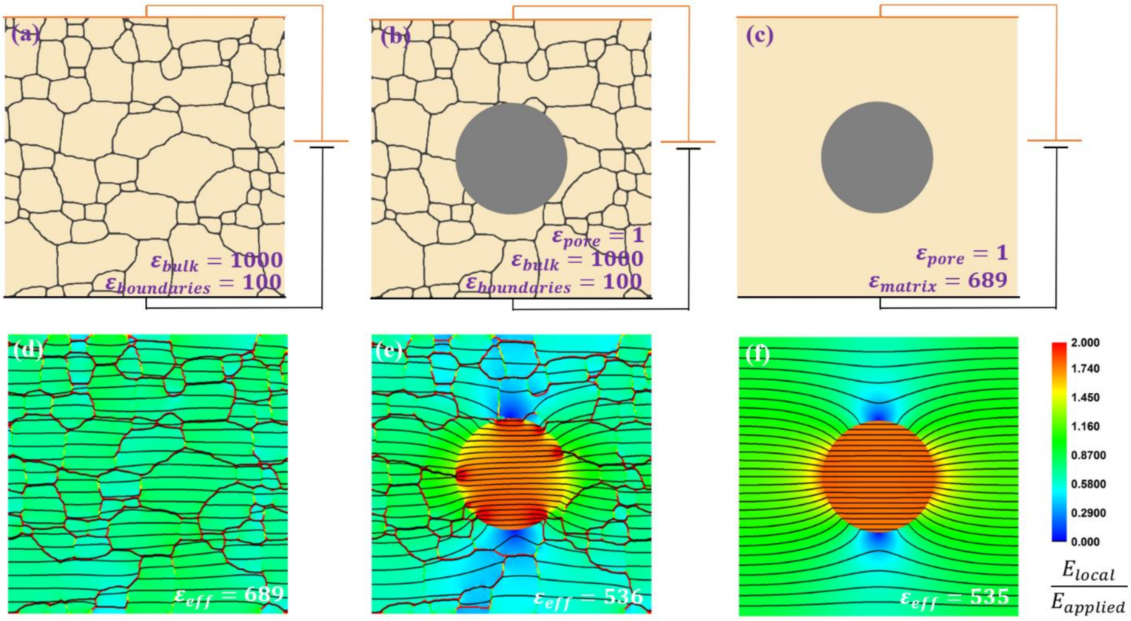

Figure S4. 2D FEM simulations for 3 equivalent ceramic systems with GS = 80 a.u.: (**a**,**d**) a dense ceramic formed by grain bulk and grain boundaries, (**b**,**f**) a porous system (tri-phase model) in which grain bulk and grain boundaries were explicitly defined and (**c**,**e**) a porous system (di-phase model) in which grains and boundaries were defined as a single phase characterized by the equivalent permittivity computed by FEM for the dense system (**a**). The local field images (**d**–**f**) are represented in color scale and the black lines are isopotential.

However, the local electric field is still well described inside the pore by the di-phase approach and, more remarkably, the two approaches provide almost the same effective permittivity of the porous structure: 536 in the case of tri-phase model and 535 in the case of di-phase model.

For fine ceramics (Figure S5) the influence of the grain boundaries become dominant and the local field on bulk is strongly reduced (blue color in Figure S5d).

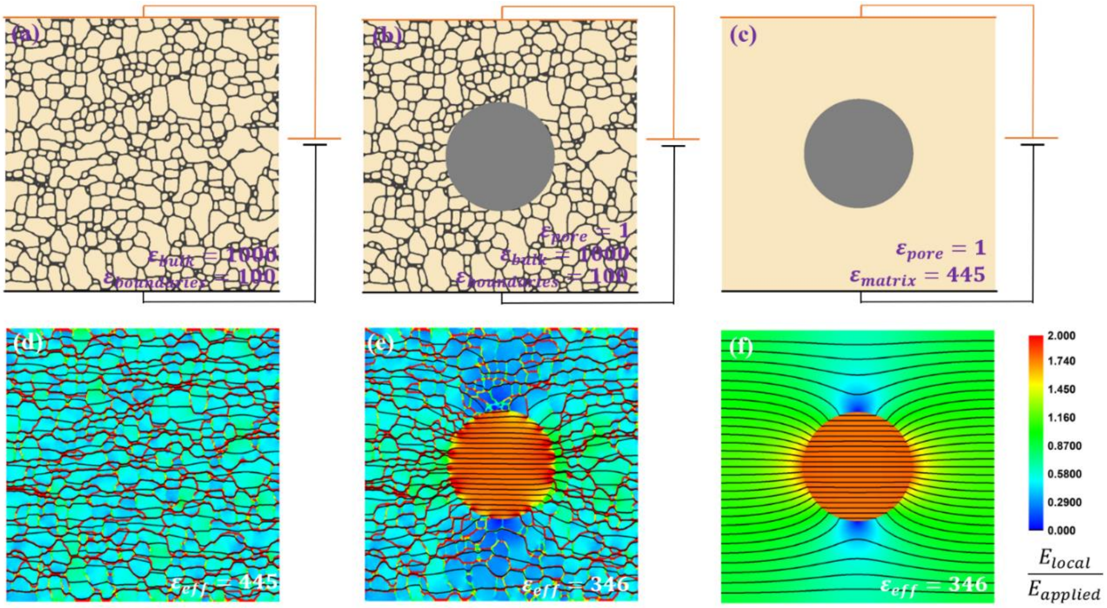

Figure S5. 2D FEM simulations for 3 equivalent ceramic systems with GS = 30 a.u.: (**a**,**d**) a dense ceramic formed by grain bulk and grain boundaries, (**b**,**f)** a porous system (tri-phase model) in which grain bulk and grain boundaries were explicitly defined and (**c**,**e**) a porous system (di-phase model) in which grains and boundaries were defined as a single phase characterized by the equivalent permittivity computed by FEM for the dense system (**a**). The local field images (**d**–**f**) are represented in color scale and the black lines are isopotential.

The calculations of the local electric field in the porous system by the two approaches revealed completely different images of the local electric field on bulk (Figure S5e,f) but still similar local electric fields inside the pore. However, the two approaches still provide almost the same value for the effective permittivity of the porous ceramic system: 346.

The modifications induced by grain boundaries in the electrostatic configurations of the porous ferroelectric ceramics can be better highlighted by the local field distributions presented in Figure S6.

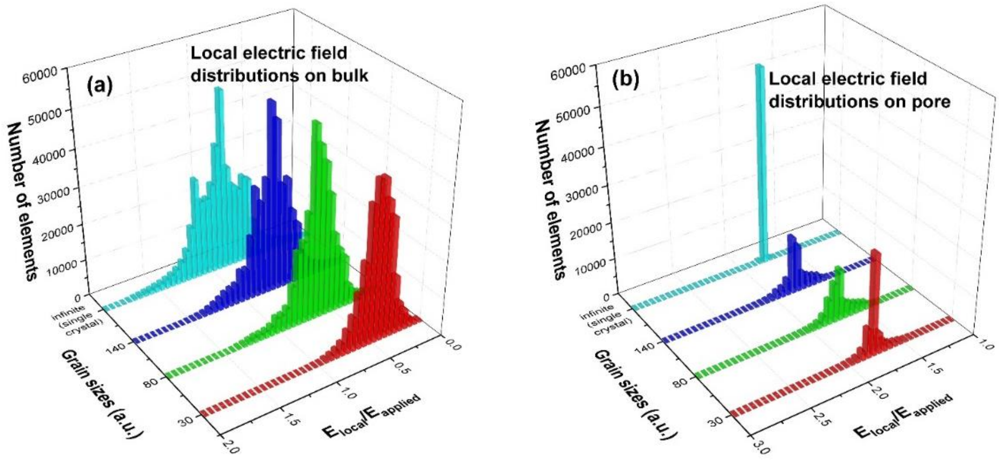

Figure S6. Local electric field distributions on grain bulk (**a**) and on the pore (**b**) calculated for different GSs.

When decreasing the GS, the average local field on bulk suffers a strong reduction, while the average local field inside the pore remains unchanged irrespective of the GS, even if the distribution suffers a small modification from the Dirac type, for large grains, to a Gaussian type, for smaller grains. Therefore, from these comparisons, the following conclusions can be derived when using a simplified di-phase composite model to simulate the properties of a porous ferroelectric ceramic: (i)

the local field inside the pore is quite well described, irrespective of the GS, at least at a qualitative level, (ii) the local electric field distribution on bulk corresponds to the effective medium replacing the real ceramic (bulk + boundaries) and this distribution might be very different from the real distributions for ceramics with reduced GS.

Even if the simulations demonstrate that the local field images provided by the two approaches are not always similar, especially in porous fine ceramics, the dependences of the effective permittivity on the grain size calculated by the two approaches (Figure S7) present a very small variation (<2%).

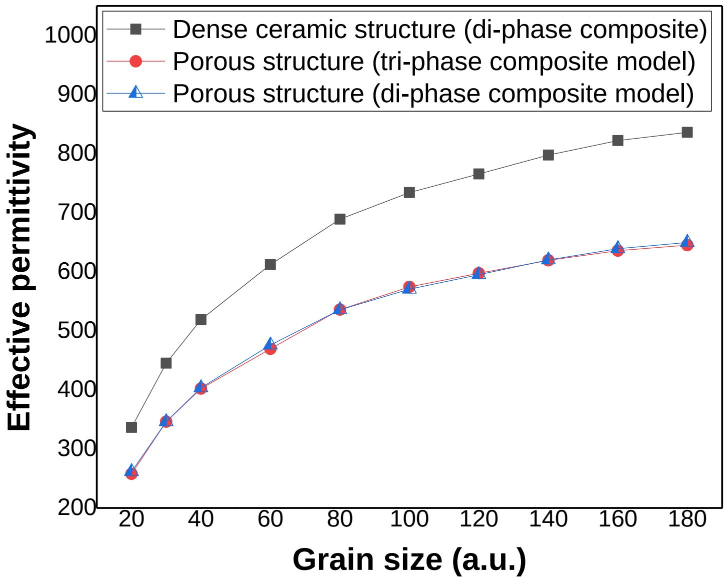

Figure S7. Simulated dependences of the effective permittivity vs. grain size for the 3 types of systems: the dense system (black curve), the tri-phase porous system (red curve) and the di-phase equivalent porous system (blue curve) in which the effective permittivity calculated for the dense structure was introduced as input as the permittivity of the dense ceramic.

The fact that the two approaches provide almost identical results can also be demonstrated by Effective Medium Theories [5]. The effective permittivity is defined as the ratio between the average electric induction and the average electric field. In particular, in the case of the porous ceramics (tri-phase composite), the effective permittivity is, Equation (S1):

$$\varepsilon_{eff} = \frac{\varepsilon_{bulk}\overline{E_{bulk}} + \varepsilon_{boundaries}\overline{E_{boundaries}} + \varepsilon_{pore}\overline{E_{pore}}}{E_{applied}} \quad \text{(S1)}$$

where, ε terms are the local permittivity values of the phases and $\bar{E}$ terms are the average electric fields on the phases. The di-phase composite model replaces the grain bulk and grain boundaries with one single continuum medium trough the relation, Equation (S2):

$$\varepsilon_{BZT}\overline{E_{BZT}} = \varepsilon_{bulk}\overline{E_{bulk}} + \varepsilon_{boundaries}\overline{E_{boundaries}} \quad \text{(S2)}$$

and the effective permittivity is calculated using the formula, Equation (S3):

$$\varepsilon_{eff} = \frac{\varepsilon_{BZT}\overline{E_{BZT}} + \varepsilon_{pore}\overline{E_{pore}}}{E_{applied}} \quad \text{(S3)}$$

As demonstrated by the Curie-Weiss extrapolations of the temperature-dependent effective permittivity graphs at high temperatures (Figure S2), the effective permittivity presents a certain reduction when reducing GS towards 0.45 μm (~20%) which can be assigned to the influence of the grain boundaries. Among the 3 cases presented in Figures S3–S5, the most appropriate to the real BZT samples presented in the manuscript is the one presented in Figure S3.

Using this study, it can be concluded that the 3D FEM approach (di-phase model) presented in the manuscript is a practical and useful way to describe the local electric fields in the BZT ceramics at a qualitative level and the effective permittivity at a quantitative level.

References

1. V. Buscaglia, S. Tripathi, V. Petkov, M. Dapiaggi, M. Deluca, A. Gajovic, Y. Ren, Average and local atomic-scale structure in $BaZr_xTi_{1-x}O_3$ (x = 0.10, 0.20, 0.40) ceramics by high-energy x-ray diffraction and Raman spectroscopy, J. Phys. Cond Matter. 26, 6, 065901 (2014)
2. A. Y. Emelyanov, N. A. Pertsev, S. Hoffman-Eifert, U. Böttger, and R. Waser, Grain-boundary effect on the Curie-Weiss law of ferroelectric ceramics and polycrystalline thin films: calculation by the Method of Effective Medium, J. Electroceram. 9, 5 (2002)
3. L. Padurariu, L. Curecheriu, V. Buscaglia, L. Mitoseriu, Field-dependent permittivity in nanostructured $BaTiO_3$ ceramics: Modelling and experimental verification, Phys. Rev. B 85, 224111 (2012)
4. R.B. Potts, Some generalized order-disorder transformations, Proc.Camb. Phil. Soc, 48, 106 (1952)
5. K.K. Karkkainen, H. Sihvola, and K. I. Nikoskinen,Effective permittivity of mixtures: numerical validation by the FDTD method, IEEE Trans. Geosci. Remote Sens. 38, 1303 (2000).
